# Prescreening for European Prevention of Alzheimer Dementia (EPAD) trial-ready cohort: impact of AD risk factors and recruitment settings

**DOI:** 10.1186/s13195-019-0576-y

**Published:** 2020-01-06

**Authors:** Lisa Vermunt, Graciela Muniz-Terrera, Lea ter Meulen, Colin Veal, Kaj Blennow, Archie Campbell, Isabelle Carrié, Julien Delrieu, Karine Fauria, Gema Huesa Rodríguez, Silvia Ingala, Natalie Jenkins, José Luis Molinuevo, Pierre-Jean Ousset, David Porteous, Niels D. Prins, Alina Solomon, Brian D. Tom, Henrik Zetterberg, Marissa Zwan, Craig W. Ritchie, Philip Scheltens, Gerald Luscan, Anthony J. Brookes, Pieter Jelle Visser, for the IMI-EPAD collaborators

**Affiliations:** 1 Department of Neurology, Alzheimer Center Amsterdam, Amsterdam Neuroscience, Amsterdam UMC, Vrije Universiteit, PO Box 7057, 1007 MB Amsterdam, The Netherlands; 2 Centre for Dementia Prevention, University of Edinburgh, Edinburgh, Scotland; 3 Department of Genetics, University of Leicester, Leicester, UK; 4 Department of Psychiatry and Neurochemistry, Institute of Neuroscience and Physiology, the Sahlgrenska Academy at the University of Gothenburg, Mölndal, Sweden; 5 Clinical Neurochemistry Laboratory, Sahlgrenska University Hospital, Mölndal, Sweden; 6 Molecular Medicine Centre, Institute of Genetics and Molecular Medicine, University of Edinburgh, Edinburgh, UK; 7 Centre de Recherche Clinique du Gérontopôle, Toulouse University Hospital, Toulouse, France; 8 Gérontopôle de Toulouse, UMR INSERM 1027, Toulouse University Hospital, Toulouse, France; 9 BarcelonaBeta Brain Research Center, Fundacio Pasqual Maragall, Barcelona, Spain; 10 Department of Radiology and Nuclear Medicine, Amsterdam Neuroscience, Amsterdam UMC, Vrije Universiteit, Amsterdam, The Netherlands; 11 Brain Research Center, Amsterdam, The Netherlands; 12 Institute of Clinical Medicine, Neurology, University of Eastern Finland, P.O. Box 1627, 70211 Kuopio, Finland; 13 Division of Clinical Geriatrics, NVS, Karolinska Institutet, Stockholm, Sweden; 14 MRC Biostatistics Unit, University of Cambridge, Cambridge, UK; 15 Department of Neurodegenerative Disease, UCL Institute of Neurology, London, UK; 16 UK Dementia Research Institute at UCL, London, UK; 17 Global Innovative Pharma Business – Clinical Sciences, Pfizer, Paris, France; 18 Department of Psychiatry and Neuropsychology, School for Mental Health and Neuroscience (MHeNS), Alzheimer Centrum Limburg, Maastricht University, Maastricht, The Netherlands

**Keywords:** Prescreening, Amyloid, Secondary prevention trials, Registries, Recruitment, Engagement, Trial-ready cohort

## Abstract

**Background:**

Recruitment is often a bottleneck in secondary prevention trials in Alzheimer disease (AD). Furthermore, screen-failure rates in these trials are typically high due to relatively low prevalence of AD pathology in individuals without dementia, especially among cognitively unimpaired. Prescreening on AD risk factors may facilitate recruitment, but the efficiency will depend on how these factors link to participation rates and AD pathology. We investigated whether common AD-related factors predict trial-ready cohort participation and amyloid status across different prescreen settings.

**Methods:**

We monitored the prescreening in four cohorts linked to the European Prevention of Alzheimer Dementia (EPAD) Registry (*n* = 16,877; mean ± SD age = 64 ± 8 years). These included a clinical cohort, a research in-person cohort, a research online cohort, and a population-based cohort. Individuals were asked to participate in the EPAD longitudinal cohort study (EPAD-LCS), which serves as a trial-ready cohort for secondary prevention trials. Amyloid positivity was measured in cerebrospinal fluid as part of the EPAD-LCS assessment. We calculated participation rates and numbers needed to prescreen (NNPS) per participant that was amyloid-positive. We tested if age, sex, education level, *APOE* status, family history for dementia, memory complaints or memory scores, previously collected in these cohorts, could predict participation and amyloid status.

**Results:**

A total of 2595 participants were contacted for participation in the EPAD-LCS. Participation rates varied by setting between 3 and 59%. The NNPS were 6.9 (clinical cohort), 7.5 (research in-person cohort), 8.4 (research online cohort), and 88.5 (population-based cohort). Participation in the EPAD-LCS (*n* = 413 (16%)) was associated with lower age (odds ratio (OR) age = 0.97 [0.95–0.99]), high education (OR = 1.64 [1.23–2.17]), male sex (OR = 1.56 [1.19–2.04]), and positive family history of dementia (OR = 1.66 [1.19–2.31]). Among participants in the EPAD-LCS, amyloid positivity (33%) was associated with higher age (OR = 1.06 [1.02–1.10]) and *APOE* ɛ4 allele carriership (OR = 2.99 [1.81–4.94]). These results were similar across prescreen settings.

**Conclusions:**

Numbers needed to prescreen varied greatly between settings. Understanding how common AD risk factors link to study participation and amyloid positivity is informative for recruitment strategy of studies on secondary prevention of AD.

**Electronic supplementary material:**

The online version of this article (10.1186/s13195-019-0576-y) contains supplementary material, which is available to authorized users.

## Background

Recruitment of participants for secondary prevention trials in Alzheimer disease (AD) is challenging, which can cause substantial delays in study completion [1, 2]. The target population for these types of clinical trials typically comprises of individuals without signs of dementia, and with evidence of amyloid pathology [3]. Clinical trial screening of these mildly symptomatic or asymptomatic participants is accompanied by large numbers of screen failures [1]. The solution may be to introduce low-burden prescreening steps, which would limit the screening efforts to individuals with an increased prospect of enrolment into the study [4–7]. However, there is little empirical evidence on prescreening for secondary prevention trials and whether the efficacy depends on recruitment setting [8–11].

The European Prevention of Alzheimer Dementia (EPAD) Registry was set up as a virtual registry from existing cohorts [12]. The purpose was to enable recruitment and preselection of individuals for participation in the EPAD longitudinal cohort study (EPAD-LCS) [13], which also serves as a trial-ready cohort for the EPAD secondary prevention trials [14]. Data on several AD-related factors were available in these existing cohorts, including age, sex, education, *APOE* genotype, family history of dementia, subjective cognitive decline (SCD), and memory tests, as well as on common exclusion criteria. Furthermore, unlike in most trials, where a participant contacts a site following advertisements, in EPAD, researchers invited participants from the cohorts in the EPAD Registry into the EPAD-LCS. This approach allowed for investigation of how AD risk factors related to the participation rate, an important consideration for the feasibility assessment of recruitment strategies. The recruitment settings linked to the registry include memory clinics, online and in-person brain research cohorts, and population-based cohorts, thereby offering the opportunity to compare them. We assessed participation rates across different recruitment settings and provide a number needed to prescreen (NNPS) to identify one eligible and amyloid-positive individual. We also tested the AD-related factors as predictors for participation in the EPAD-LCS and for amyloid positivity.

## Methods

### Population

The analysis included participants from the first four cohorts that were linked to the EPAD Registry. The *French Trial Registry* in Toulouse selected patients referred by GPs and self-referral from memory clinics [15]. Inclusion criteria were as follows: interest in clinical trials, available study partner, and no obvious exclusion criteria for clinical trials. Data from 195 participants without dementia, with visits between July 2016 and February 2018, had been linked to the EPAD Registry. The *ALFA Study* included cognitively unimpaired individuals who expressed interest in participating in AD research and data of 2595 participants aged over 50 years, with first visits in 2013 and 2014, were linked to the EPAD Registry [16]. *Generation Scotland* (GS) was a population-based study which collected data between 2006 and 2011 in Scotland on randomly drawn individuals with a relative to co-enrol [17]. Its aim was to create a resource of human biological samples and information for medical research, and data on 13,681 participants aged over 50 years, without a known diagnosis of dementia, were linked to the EPAD Registry. The *pilot ‘hersenonderzoek.nl’ (pilotHO.nl)* was a web-based registry with the aim of recruiting people from the general public for brain research and ran from Sept 2016 to Sept 2017 when the final version of the registry was launched. This pilot registry had 412 participants, age over 50 years and without a self-reported diagnosis of dementia, linked to the EPAD Registry.

### EPAD Registry selection and prescreening process

The enrolment process for the EPAD-LCS consisted of four steps. In step 1, participants were preselected from the four cohorts using algorithms in the EPAD Registry online tool [18], based on different combinations of age, sex, diagnosis of mild cognitive impairment (MCI), *APOE* genotype, SCD, memory test scores, and/or family history for dementia, available in the parent cohort (Table 1). Flexible algorithms were tailored to each of the cohorts, and adjusted if the number of individuals meeting the algorithmic criteria was low. The algorithms selected individuals older than 50 years across an AD dementia risk spectrum [13]. These included those with low and medium risk for AD to reach the recruitment targets for the study, as well as to avoid AD risk status disclosure by invitation. In step 2, the cohorts’ investigators checked eligibility of selected individuals, using data from their databases. These criteria included the EPAD in- and exclusion criteria, which involve absence of disorders that could interfere with trial participation, absence of dementia, and openness to potentially participate in intervention studies and receive disclosure [13]. In three of the cohorts, preselected individuals were then approached by telephone for participation. The population-based cohort GS sent an opt-in letter. In step 3, the EPAD sites performed a telephone screen to check eligibility among those who expressed interest in participating. Prescreen failures during the first 3 steps were categorised as ‘matching an exclusion criterion’, ‘no interest in participation in the study’, ‘not returning the opt-in letter’, ‘other reason, not specified’ [12]. In step 4, participants visited a site and enrolled in the EPAD-LCS for a screening/baseline visit, after which eligibility was confirmed and amyloid status was determined [13].

**Table 1:** Baseline available data and characteristics of cohorts

	Toulouse Registry	ALFA	Generation Scotland	pilotHO.nl
Setting	Memory clinic	In-person research cohort	Population-based	Online research cohort
*N*	195	2589	13,681	412
Age, year	68 (7)	60 (6)	64 (9)	65 (9)
Male, *n* (%)	56 (29%)	962 (37%)	5399 (39%)	155 (38%)
Highly educated, *n* (%) (*n* = 15,239)^*^	97 (60%)	1225 (47%)	4860 (40%)	313 (77%)
*APOE* ɛ4 genotype, *n* (%) (*n* = 16,185)	NA	872 (34%)	3695 (28%)	84 (31%)
Family history for dementia, *n* (%) (*n* = 16,844)	131 (71%)	2470 (95%)	1386 (10%)	193 (50%)
Subjective cognitive decline, *n* (%) (*n* = 3175)^^^	151 (83%)	312 (12%)	NA	81 (20%)
% low memory, *n* (%) (*n* = 16,420)^$^	17 (15%)	242 (9%)	1684 (12%)	20 (9%)
Diagnosed with MCI, *n* (%)^#^	13 (7%)	0	3 (0%)	4 (1%)
Estimated amyloid-positive individuals based on [4], taking into account age-bins, *n* (%) ^&^	~ 40 (22%)	~ 430 (17%)	~ 2680 (20%)	~ 80 (20%)

Legend: ^*^ high education: Toulouse Registry: > = 14 years; ALFA Study: > = 14 years; GS: > = 14 years; pilotHO.nl: > = 6 on the Verhage scale. ^^^SCD: Toulouse Registry: physician diagnosis and MCI patients excluded; ALFA Study: memory complaints question; pilotHO.nl: questions on memory complaints with worries; ^$^Low memory delayed recall *z*-score < − 1.28: Toulouse Registry: FCSRT delayed recall, normalised by formula (score-11)/2, at raw score cut-off < 9; ALFA Study: memory binding test, normalised to sample, at raw score cut-off < 18; GS: Wechsler logical memory - delayed recall was normalised, at raw score cut-off < 9; pilotHO.nl: online Muistikko-test, normalised to sample, at raw score cut-off < 9. ^#^MCI: Toulouse Registry: physician diagnosis; pilotHO.nl and Generation Scotland self-report. In the ALFA study no MCI patients were enrolled. ^&^Formula based on Janssen et al. [4] (*N* age bin 50 to 57.5 years) × 12.9% + (*N* age bin 57.5 to 62.5 years) × 15.8% + (*N* age bin 62.5 to 67.5 years) × 19.2% + (*N* age bin 67.5 to 72.5 years) × 23.1% + (*N* age bin 72.5 to 77.5 years) × 27.6% + (*N* age bin 77.5 to 82.5 years) × 32.6% + (*N* age bin 82.5 years and older) × 38%

### Data collected as part of the EPAD-LCS

From the EPAD-LCS baseline visit, we used clinical information, i.e., the CDR sum of boxes (CDR-SOB) and Mini-Mental State Examination (MMSE); structural MR imaging visual rating scales, i.e., the medial temporal atrophy scale (MTA) mean score and Fazekas deep score of white matter hyperintensities. From the cerebrospinal fluid (CSF) analysis, we used Elecsys Aβ1–42, total tau, and phosphorylated tau values, and from the blood analyses, for some participants, *APOE* ɛ4 genotype. For a full description of the EPAD-LCS protocols, we refer to [13].

### Predictors

The predictors as collected in the cohorts linked to the Registry were as follows: age, sex, education level (low to normal or high), *APOE* genotype (ɛ4 non-carrier or carrier), presence of family history for dementia, presence of SCD, and a low score on a delayed recall memory test (*z*-score < − 1.28, details on definitions of variables Supplement, legend Table 1). All cohorts had data available on demographics. SCD data was present in all cohorts, except GS. *APOE* genotype was available in the ALFA Study, GS, and a subset of pilotHO.nl. Family history and memory test scores were available for all participants of the ALFA Study and GS, and for the majority in the Toulouse Registry and pilotHO.nl. The definitions of the predictors were as follows: high education was 14 years or more in Toulouse Registry, the ALFA Study, and GS, and in pilotHO.nl a score of 6 or more on the Verhage scale, equivalent to college or university level [19]. Subjective cognitive decline: presence of memory complaints in the absence of impairment on cognitive tests (Toulouse Registry); a positive answer on the question whether the participant memory had complaints (ALFA study), a positive answer on the questions whether the participant memory had complaints and worries about their memory (pilotHO). Low memory delayed recall *z*-score < − 1.28 on the FCSRT delayed recall (Toulouse), the memory binding test (ALFA study), the Wechsler logical memory - delayed recall (GS), and the Muistikko-test (pilotHO).

### Outcomes

The first outcome measure was enrolment into the EPAD-LCS, indicating participation in a screening/baseline visit. The second outcome was amyloid positivity, defined as CSF Aβ1–42 below 1098 pg/mL [20–22], for participants who completed and passed the eligibility checks of the EPAD-LCS screening visit.

### Statistical analysis

Participation rate was defined as the percentage of individuals who underwent the EPAD-LCS screening visit out of the individuals approached for participation in the EPAD-LCS. The NNPS was defined as the ratio between the number of individuals contacted for participation and the number of individuals that passed baseline visit classified as amyloid positive. The number needed to screen (NNS) was the ratio between the number of individuals with baseline data and the number of individuals that passed screening visit who were classified as amyloid positive. To test the association between AD risk factors (predictors) and participation into the EPAD-LCS, and among those enrolled, between AD risk factors and amyloid positivity, we applied univariate logistic mixed models with a random term for cohort and fixed term for the predictor. Age was centred at 65. Explorative analyses included analyses stratified now by cohort using univariate logistic regression models. Additionally, as a second step, all significant predictors for either of the two outcomes were combined in two final multivariate models to summarise the results. Statistical analyses were performed in R version 3.4.2, using packages ‘lme4’ and ‘lmerTest’ [23, 24].

## Results

**Fig. 1:**
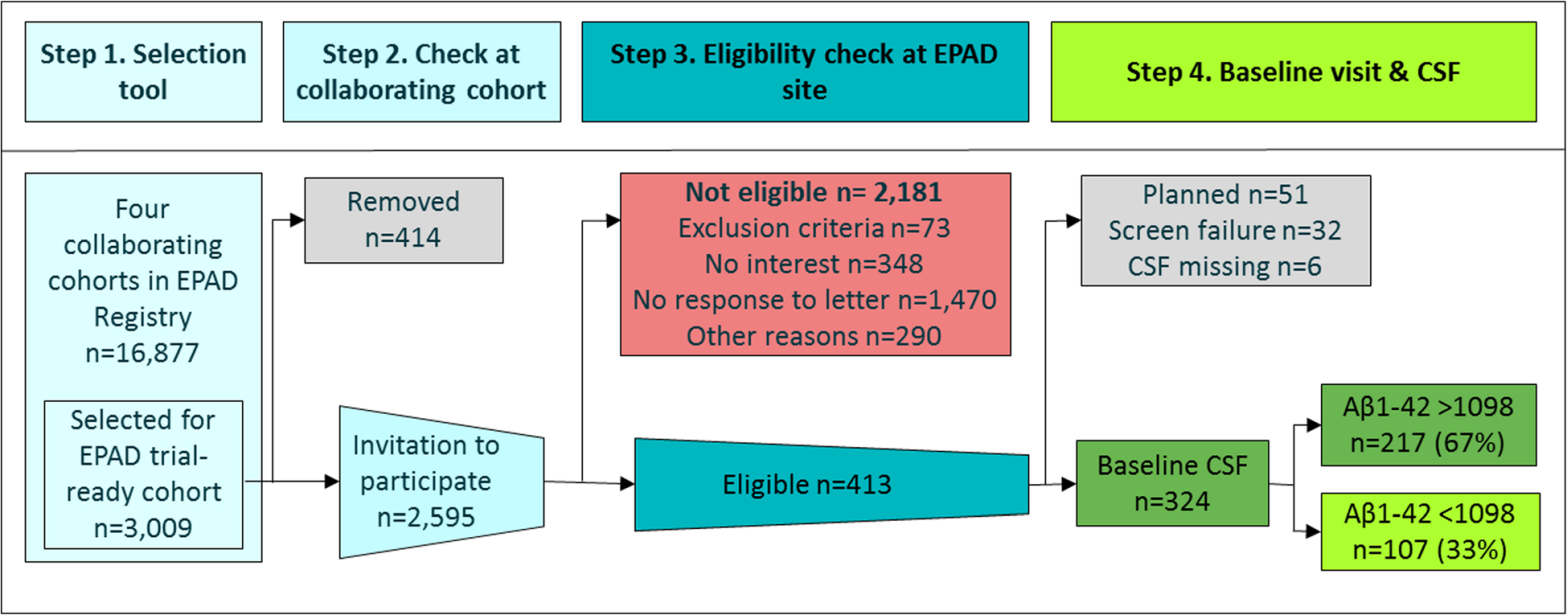
Prescreening to enrolment: flow from EPAD Registry to EPAD trial-ready cohort. *Legend*: CSF = cerebrospinal fluid; EPAD = European Prevention of Alzheimer Dementia

The four cohorts linked to the EPAD Registry included 16,877 participants. The participants were on average 64 (SD = 8) years old and 39% were male, and expected amyloid positivity was calculated to be 19% based on a published meta-analysis [4] (Table 1). Figure 1 and Table 2 describe the recruitment flow of participants to enrolment and amyloid measurement in the EPAD-LCS between May 2016 and March 2018. Table 3 presents clinical, imaging, and CSF markers of the EPAD-LCS baseline visit for participants recruited from each of the cohorts, stratified by amyloid status.

**Table 2:** Recruitment flow from EPAD Registry by recruitment setting

		Cohorts				Total
		Toulouse Registry	ALFA Study	Generation Scotland	pilotHO.nl	
	Setting	Memory clinic	In-person research cohort	Population-based	Online research cohort	
Step 1	Selection by PREPAD tool	*169*	*618*	*1947*	*275*	*3009*
Step 2	Not eligible based database check	11	347	1	55	414
	• Exclusion criterion	10	110	1	29	150
	• Other	1	237	0	26	264
	Number selected for step 3 (% from step 1)	*158 (93%)*	*271 (44%)*	*1946 (100%)*	*220 (80%)*	*2595 (86%)*
Step 3	Not eligible after contacting participant	65	119	1879	119	2182
	• No interest	64	24	178	83	349
	• No response to letter	NA	NA	1470	NA	1470
	• Exclusion criterion	0	37	12	24	73
	• Other	1	58	219	12	290
	Number selected for step 4 (% from step 3)	*93 (59%)*	*152 (56%)*	*67 (3%)*	*101 (46%)*	*413 (16%)*
Step 4	EPAD-LCS screening visit	70	137	67	88	362
	Eligible & CSF Aβ1–42 analysed	*64*	*124*	*61*	*75*	*324*
	• CSF Aβ1–42 < 1098 pg/mL (positivity)	23 (36%)	36 (29%)	22 (36%)	26 (35%)	107 (33%)
	Number needed to screen	3.0	3.8	3.0	3.4	3.4
	Number needed to prescreen	6.9	7.5	88.5	8.5	24.3

Legend: in italic the total number of the subset on which percentages are based. Number of individuals unless otherwise specified. *CSF* cerebrospinal fluid, EPAD-LCS v500 is the currently available data, quality checked at data lock. *N* = 51 EPAD screening visit details not yet available. *N* = 5 CSF results missing. *N* = 32 screen failure: 11× other disease/incidental findings/CDR > =1, 18× procedures not possible, 3× investigator decision/no reason provided/no contact possible. Number needed to screen = *N* CSF Aβ1–42 positive in step 4 / *N* eligible and CSF Aβ1–42 analysed in step 4, while the number needed to prescreen = *N* CSF Aβ1–42 positive in step 4/ *N* selected for step 3

**Table 3:** Included participants in EPAD Longitudinal cohort study per recruitment setting

	Toulouse Registry		ALFA Study		Generation Scotland		pilotHO.nl	
	CSF Aβ +ve	CSF Aβ normal	CSF Aβ +ve	CSF Aβ normal	CSF Aβ +ve	CSF Aβ normal	CSF Aβ +ve	CSF Aβ normal
*n*	23	41	36	88	22	39	26	49
Age, year	71 (5)	67 (8)^^^	64 (6)	64 (5)	71 (3)	67 (5)^#^	68 (6)	66 (7)
Male, *n* (%)	4 (17%)	17 (41%)	23 (64%)	41 (47%)	14 (61%)	23 (57%)	14 (52%)	19 (37%)
MMSE (30–0)	28.0 (2.1)	28.8 (1.7)	28.6 (1.1)	28.7 (1.6)	28.1 (1.6)	28.8 (1.4)	28.4 (1.5)	29.1 (1.3)
CDR-SOB (0–18)	0.74 (0.7)	0.34 (0.5)^^^	0.10 (0.3)	0.05 (0.2)	0.15 (0.3)	0.06 (0.2)	0.06 (0.2)	0.02 (0.1)
CSF Aβ1–42, pg/mL	756 (195)	1613 (361)^#^	823 (191)	1696 (519)^#^	748 (251)	1769 (411)^#^	846 (217)	1788 (443)^#^
CSF P-tau, pg/mL	29 (15)	18 (4.9)^$^	21 (15)	17 (7)	19 (9)	21 (12)	21 (10)	17 (5)
CSF T-tau, pg/mL	305 (125)	210 (53)^$^	223 (132)	209 (79)	211 (81)	249 (115)^*^	240 (101)	206 (58)
MTA (0–4)	0.4 (0.5)	0.2 (0.4)	0.2 (0.4)	0.1 (0.3)	0.4 (0.6)	0.1 (0.4)	0.2 (0.4)	0.2 (0.4)
Fazekas (0–3)	1.1 (0.7)	0.8 (0.7)	1 (0.6)	0.8 (0.6)	1.1 (0.9)	0.7 (0.7)^^^	0.9 (0.8)	0.9 (0.7)

Legend: *MMSE* Mini-Mental State Examination, *CDR-SOB* Clinical Dementia Rating scale- Sum Of Boxes, *CSF* cerebrospinal fluid, *P-tau* phosphorylated tau, *T-tau* total tau, *MTA* medial temporal lobe atrophy. Mean (SD) unless otherwise specified. ^*^One outlier at t-tau 792 and p-tau 81. Undetectably low p-tau and t-tau was set at the detection border of 8 and 80 respectively, Aβ1–42 was extrapolated. Raw *p* < 0.05 = ^^^; *p* < 0.01 = ^$^, *p* < 0.001 = ^#^

From the EPAD Registry, 3009 individuals were preselected for participation in the EPAD-LCS and 2595 individuals were contacted, of whom 413 (16%) agreed to participate and were eligible for the EPAD-LCS screening visit. To prevent contacting individuals matching exclusion criteria for the EPAD-LCS, most cohorts conducted a database check. This was most efficient in the Toulouse registry (100%). Of the individuals with exclusion criteria in the ALFA Study and in pilotHO.nl, 75% (110/147) and 55% (24/53) were found during the database check, respectively. Participation rate varied by setting; in the Toulouse Registry, it was 59%, in the ALFA Study 56%, in GS 3%, and in pilotHO.nl 46%. The primary reasons for not participating were not returning the opt-in leaflet (67%), no interest (16%), and other reasons (13%). Of the 324 participants who had passed the eligibility checks during EPAD-LCS screening visit and had their amyloid status available, 107 (33%) participants were amyloid positive. The total number of amyloid-positive individuals was similar between cohorts (Toulouse Registry *n* = 23, ALFA Study *n* = 36, GS *n* = 22, pilotHO.nl *n* = 26). However, the NNPS to find one eligible amyloid-positive participant varied; in the Toulouse Registry, it was 6.9, in the ALFA Study 7.5, in GS 88.5, and in pilotHO.nl 8.4. Among individuals enrolled in the EPAD-LCS, the NNS in order to find one amyloid-positive individual passing the screening visit was between 3.0 and 3.8 in all settings (Table 2).

### Predictors for participation rate

**Fig. 2:**
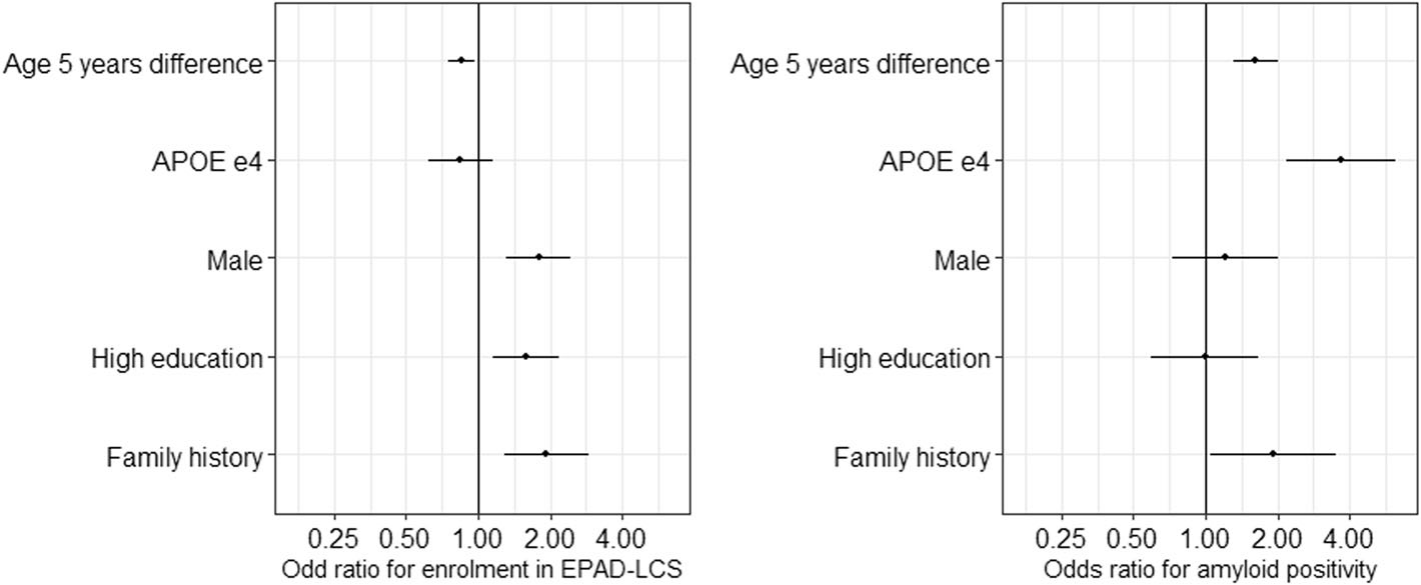
Multivariate model for enrolment and amyloid positivity. Legend: EPAD-LCS = EPAD longitudinal cohort study (trial-ready cohort). *APOE* = apolipoprotein E gene. Shown effect sizes are: Age per 5 years older at baseline, APOE ɛ4 in contrast to no APOE ɛ4, male in contrast to female, highly educated in contrast to low or normal level educated, family history for dementia positive in contrast to family history for dementia reported

The AD risk factors that were univariately associated with participation in the EPAD-LCS, for all cohorts combined, were lower age (odds ratio (OR): age = 0.97 [0.95–0.99]), high education level (OR = 1.64 [1.23–2.17]), male sex (OR = 1.56 [1.19–2.04]), and family history of dementia (OR = 1.66 [1.19–2.31], Table 4, for AUCs Additional file 1: Table S2). In single cohorts, participation rates in the Toulouse Registry were predicted by SCD (OR = 0.29; [0.09–0.76]), in the ALFA Study by male sex (OR = 2.03 [1.24–3.35]), in GS by male sex (OR = 1.81 [1.11–3.01]), high education (OR = 2.20 [1.34–3.59], and family history (OR = 2.95 [1.73–4.91], and in pilotHO.nl by age (OR = 0.96 [0.93–1.00]). As a next step, we combined the predictor variables age, sex, education, family history, and *APOE* in a multivariate model (Fig. 2, Additional file 1: Table S1 and Table S3). Study enrolment was still associated with age, sex, education, and family history (*n* with all variables = 2322).

**Table 4:** Univariate logistic regression for enrolment and CSF Aβ1–42 positivity in whole sample and stratified by recruitment setting

Sample size	Total		Toulouse Registry		ALFA Study		Generation Scotland		pilotHO.nl	
	*n* = 2595	*n* = 324	*n* = 158	*n* = 64	*n* = 271	*n* = 124	*n* = 1947	*n* = 61	*n* = 220	*n* = 75
Outcome	Enrolment^*^	*CSF Aβ +ve*^*^*^	Enrolment^*^	*CSF Aβ +ve*^*^*^	Enrolment^*^	*CSF Aβ +ve*^*^*^	Enrolment^*^	*CSF Aβ +ve*^*^*^	Enrolment^*^	*CSF Aβ +ve*^*^*^
Aged over 70 years Old	*0.97 (0.95–0.99)*	*1.06 (1.02–1.10)*	0.99 (0.94–1.03)	*1.10 (1.01–1.20)*	0.99 (0.95–1.03)	1.01 (0.94–1.08)	0.97 (0.93–1.01)	*1.23 (1.08–1.45)*	*0.96 (0.93–1.00)*	1.03 (0.97–1.11)
Male	*1.56 (1.19–2.04)*	1.28 (0.81–2.04)	1.17 (0.58–2.42)	*0.30 (0.08–0.96)*	*2.03 (1.24–3.35)*	2.03 (0.92–4.60)	*1.81 (1.11–3.01)*	1.35 (0.47–4.08)	1.13 (0.66–1.94)	2.01 (0.77–5.36)
Highly educated	*1.64 (1.23–2.17)*	0.89 (0.56–1.42)	1.44 (0.69–2.98)	0.72 (0.25–2.13)	1.42 (0.87–2.31)	1.10 (0.50–2.39)	*2.20 (1.34–3.59)*	0.66 (0.22–1.88)	1.33 (0.67–2.67)	0.75 (0.24–2.51)
*APOE* ɛ4 genotype	0.95 (0.70–1.28)	*2.99 (1.81–4.94)*	NA	*6.42 (1.93–24.1)*	0.68 (0.41–1.10)	1.72 (0.79–3.86)	1.37 (0.84–2.25)	*7.20 (2.2–28.77)*	0.92 (0.49–1.72)	*3.34 (1.22–9.48)*
Family history of Dementia	*1.66 (1.19–2.31)*	1.58 (0.83–3.00)	1.04 (0.50–2.15)	0.95 (0.31–2.98)	1.12 (0.38–3.23)	NA^#^	*2.95 (1.73–4.91)*	2.90 (0.97–8.96)	1.27 (0.73–2.22)	1.94 (0.68–6.09)
Subjective cognitive decline	0.86 (0.58–1.27)	1.51 (0.88–2.61)	*0.29 (0.09–0.76)*	2.93 (0.67–20.6)	0.79 (0.41–1.55)	1.15 (0.38–3.22)	NA	NA	1.16 (0.62–2.15)	1.73 (0.62–4.79)
Low memory score	0.84 (0.60–1.17)	1.47 (0.82–2.61)	0.63 (0.21–1.87)	*18.90 (2.87–377)*	0.95 (0.56–1.64)	1.29 (0.55–2.96)	0.78 (0.44–1.31)	0.95 (0.28–3.02)	0.91 (0.29–2.83)	0.58 (0.03–4.98)

Odds ratio (95% CI); *CSF* cerebrospinal fluid. Italics is significant *p* < 0.05. *Odds ratio for participating baseline/screening visit after invitation. ^Odds ratio for amyloid positivity among those included in EPAD-LCS. ^#^Infinite, not possible to calculate a value

### Predictors for amyloid positivity

Among all individuals enrolled in EPAD-LCS, amyloid positivity was univariately predicted by older age (OR = 1.06 [1.02–1.10]) and carrying an *APOE* ɛ4 allele (OR = 2.99 [1.81–4.94]) (Table 4, for AUCs, Additional file 1: Table S2). In individual cohorts, amyloid positivity in the Toulouse Registry was predicted by higher age (OR = 1.10 [1.01–1.20]), gender (male OR = 0.30 [0.08–0.96]), *APOE* ɛ4 (OR = 6.42 [1.93–24.1]), and low memory (OR = 18.90 [2.87–377]; in the ALFA Study by none; in GS by higher age (OR = 1.23 [1.08–1.45]) and *APOE* ɛ4 (OR = 7.20 [2.20–28.77]); and in pilotHO.nl by *APOE* ɛ4 (OR = 3.34 [1.22–9.48]). In the multivariate model, including predictor variables age, sex, education, family history, and *APOE*, amyloid status was predicted by age, *APOE* ɛ4, and weakly by family history (*p* = 0.03, *n* with all variables = 322, Fig. 2, Additional file 1: Table S1 and Table S3).

## Discussion

Across settings, participation rates varied, while predictors for participation into the trial-ready cohort and amyloid positivity were comparable. Among those contacted for participation, enrolment was higher for individuals who were younger, more educated, or males or had a family history of dementia, while amyloid positivity in the trial-ready cohort was only associated with being older and carrying an *APOE* ɛ4 allele.

The NNPS to find one amyloid-positive eligible participant in the population-based Generation Scotland study was ten times higher than for those cohorts focussed on brain disorders, which may be explained by their willingness to take part in an AD study [25]. Generation Scotland study visits have been completed, and the time between the last Generation Scotland study visit and EPAD recruitment was also longer than for the other cohorts. In addition, an opt-in letter was sent to Generation Scotland participants, while other cohorts contacted individuals by telephone, which may have lowered the response [26]. Moreover, the EPAD study site was at a travel time of 1–3 h from the recruitment region. Finally, the cohorts from the other settings excluded persons with known exclusion criteria beforehand based on data from their cohort database, which may have decreased later stage prescreen failures. Still, the number of participants recruited of the large population-based Generation Scotland cohort was comparable to the bespoke cohorts, suggesting that there is scope and willingness within these types of cohorts to participate in dementia-related intervention studies.

Lower participation at older ages and higher participation for both highly educated participants and those with a family history of dementia are in line with studies with dementia patients and online registers [9, 10, 27–29]. Barriers for older individuals to participate may include morbidities, difficulties to travel, and not having a study partner. The higher participation rate of males was unexpected, as many research studies have lower male than female participation [9, 10, 30, 31].

The predictors for amyloid positivity, i.e. age and *APOE*, were as expected and in line with previous studies, including an EPAD-LCS full dataset analysis [4, 6, 32, 33]. Low memory scores, in contrast, were only a significant predictor for amyloid positivity in the memory clinic cohort and the presence of SCD did not predict amyloid positivity in our sample. As low memory scores were the best predictor for amyloid positivity in the memory clinic setting, memory tests may form a useful prescreen in this situation. An explanation for the discrepancy with previously reported associations of these factors with amyloid status could be the non-standardised test data and could possibly show better predictive effects with the use of tailored sensitive tests and questionnaires [9, 11, 32, 34–37].

The prevalence of amyloid positivity in those enrolled in the EPAD-LCS was 33%. This prevalence was enriched around 1.5 times compared to the estimated prevalence in the whole cohorts based on a meta-analysis of prevalence in cognitively normal individuals [4]. The limited increase in prevalence of amyloid positivity could be explained by the fact that the variables available for prescreening each have a modest predictive accuracy for amyloid positivity [4, 6]. Another explanation is that low- and intermediate-risk individuals were selected from the cohorts in order to prevent risk disclosure by invitation and to have sufficient enrolment in the EPAD-LCS.

An advantage of our approach compared to other recruitment strategies such as media campaign advertisement is that the use of existing data helped to exclude individuals with known exclusion criteria for secondary prevention trials. However, no direct comparison of efficiency relative to other prescreening strategies (e.g. advertising) could be made. A disadvantage of our approach is that consent to re-contact needs to be present in the cohorts and some costs are involved in the prescreening. In addition, cohorts become depleted, as shown for the smaller cohorts in our study. Future projects could involve direct comparisons between recruitment strategies and focus on cost and effort monitoring and comparison. Another important factor when recruiting from collaborating studies, as well as in the gathering of a ‘trial-ready cohort’ is the aspect of time and cohort maintenance costs of both the recruitment cohorts and EPAD-LCS, but substantial. As AD is a progressive disorder, the time between testing in a parent cohort and time of selection may be important. Future work on the EPAD-LCS and similar projects needs to optimise the costs and efforts of maintaining a trial ready cohort. This should also involve monitoring the rate at which individuals become ineligible over time, for example because they develop comorbidities that are exclusion criteria.

A limitation is that the analyses were done with the risk factors available in each cohort, such that not all risk factors were available in all cohorts for all individuals. Also, the use of the available data and adaptation to local standard procedures meant that there was variability in the operationalisation of variables. Secondly, algorithms for preselection in the EPAD Registry tool included predictor variables of the current study. Still, that is unlikely to influence the association between each of the risk factors and participation rate as multivariate models yielded similar results. Additionally, cohorts were different from each other in more than one factor, such as sample size, population characteristics, and communication style. Therefore, differences in recruitment rate may be explained by several factors. Despite the differences, participation rate was associated with similar AD risk factors across cohorts. Finally, we have now studied the participation in a trial-ready cohort, but enrolment into an actual clinical trial might give different results, depending on study-specific in- and exclusion criteria and trial design [38]. Strengths of our study are the prospective prescreening and the large sample in which amyloid-testing was performed.

Our comparison of common AD risk factors for their association with participation rate and amyloid positivity has several implications for prescreening strategies for secondary prevention trials aimed at individuals with amyloid pathology. Age was a relatively strong predictor for amyloid positivity. However, we also showed that elderly individuals were less likely to participate in the study, which would limit the prescreening efficiency of age for amyloid positivity. Therefore, addressing barriers for older individuals to participate could increase recruitment of eligible participants [29, 39]. Carrying an *APOE* ɛ4 allele was also a strong predictor of amyloid status, but as published before, the disadvantage is that around 40% of amyloid-positive individuals are *APOE* ɛ4 non-carriers [40]. The prevalence of *APOE* ɛ4 positivity is around 20–30% and this may therefore not be optimal for prescreening in a small cohort. Disclosure of genotype could also be an issue [10, 41]. These limitations may be overcome by using a family history for dementia as a prescreener. The advantage of this risk factor is the association with a greater enrolment rate, but the disadvantage is that its association with amyloid positivity is weak and the prevalence in the general population low. Subtle memory decline or concerns were not a useful prescreen for amyloid status in our study, but more specific tests or questionnaires may perform better [11, 42, 43]. A promising alternative may be blood tests for amyloid [5, 44, 45]. With a sensitive threshold, such a test has the advantage to more effectively prescreen relatively younger individuals, who often comprise a large part of a registry population and are more likely to participate, but have a low prevalence of amyloid pathology.

## Conclusions

We found that enrolment rates show major differences between cohorts, although predictors for participation were similar. The provided NNPS to find one eligible amyloid-positive participant are indicators that future recruitment strategies can relate to. The findings highlight considerations of clinical trial investigators, balancing a gain in the ease of recruitment with potentially reducing the generalizability of the trial. Measures to increase efficiency for recruitment for secondary prevention trials may include using prospective registries with continuous enrolment of participants, adding a prescreening step with sensitive measures, such as a blood test, and addressing barriers for older and lower-educated individuals to participate.

## Supplementary information

Additional file 1: **Table S1**. Multivariate logistic regression for enrolment and CSF Aβ1–42 positivity in whole sample. **Table S2.** AUC for binominal ROC curves of Table 4. **Table S3.** AUC on multivariate model Fig. 2. (DOCX 23 kb)

## Data Availability

The EPAD data used in this analysis will be made available on an open-access platform in due course. (www.ep-ad.org for updates).

## Abbreviations

AD: Alzheimer disease
*APOE*: *Apolipoprotein E*
CDR-SOB: Clinical Dementia Rating Scale Sum of Boxes
EPAD: European Prevention of Alzheimer Dementia
EPAD-LCS: EPAD longitudinal cohort study
GS: Generation Scotland
MMSE: Mini-Mental State Examination
MTA: Medial temporal lobe atrophy
NNPS: Number needed to prescreen
NNS: Number needed to screen
P-tau: Phosphorylated tau
SCD: Subjective cognitive decline
T-tau: Total tau
