# Supplementary material for: Prescreening for European Prevention of Alzheimer Dementia (EPAD) trial-ready cohort: impact of AD risk factors and recruitment settings
Source: Alzheimers Res Ther. 2020 Jan 6;12:8. doi: 10.1186/s13195-019-0576-y (PMC6945608; doi:10.1186/s13195-019-0576-y)
Supplement: Supplementary file 1 — Table S1. Multivariate logistic regression for enrolment and CSF Aβ1–42 positivity in whole sample. Table S2. AUC for binominal ROC curves of Table 4. Table S3. AUC on multivariate model Fig. 2. (DOCX 23 kb) [file 13195_2019_576_MOESM1_ESM.docx]

**Supplemental information**

**Table S1. Multivariate logistic regression for enrolment and CSF Aβ1-42 positivity in whole sample**

|  | **Enrolment** | | | | | **CSF Aβ1-42 positivity** | | | | |
| --- | --- | --- | --- | --- | --- | --- | --- | --- | --- | --- |
| **Sample size** |  | | **N=2322** | |  |  | | **N=322** | |  |
| **Outcome** | **Univariate** | **Multivariate** | | **Multivariate**  **p-values** | | **Univariate** | **Multivariate** | | **Multivariate**  **p-values** | |
| **Age years** | **0.97**  **(0.95-0.99)** | **0.97**  **(0.95-0.99)** | | 0.011 | | **1.06**  **(1.02-1.10)** | **1.10**  **(1.05- 1.15)** | | <0.001 | |
| ***APOE* ɛ4 genotype** | 0.95  (0.70-1.28) | 0.85  (0.62-1.15) | | 0.291 | | **2.99**  **(1.81-4.94)** | **3.69**  **(2.18-6.24)** | | <0.001 | |
| **Male** | **1.56**  **(1.19-2.04)** | **1.79**  **(1.31-2.45)** | | <0.001 | | 1.28  (0.81-2.04) | 1.20  (0.72-2.00) | | 0.476 | |
| **Highly educated** | **1.64**  **(1.23-2.17)** | **1.58**  **(1.15-2.17)** | | 0.005 | | 0.89  (0.56-1.42) | 0.99  (0.60-1.66) | | 0.977 | |
| **Family history of dementia** | **1.66**  **(1.19-2.31)** | **1.93**  **(1.29-2.88)** | | 0.001 | | 1.58  (0.83-2.61) | **1.91**  **(1.05-3.49)** | | 0.034 | |

*Legend:* Odds ratio (95% confidence intervals). CSF = cerebrospinal fluid. APOE = Apolipoprotein E gene. Shown effect sizes are: Age per 5 years older at baseline, APOE ɛ4 in contrast to no APOE ɛ4, male in contrast to female, highly educated in contrast to low or normal level educated, family history for dementia positive in contrast to family history for dementia reported.

**Table S2. AUC for binominal ROC curves of table 4**

|  | **Toulouse Registry** | | **ALFA Study** | | **Generation Scotland** | | **plotHO.nl** | |
| --- | --- | --- | --- | --- | --- | --- | --- | --- |
| **Sample size** | **n=158** | **n=64** | **n=271** | **n=124** | **n=1,947** | **n=61** | **n=220** | **n=75** |
| **Outcome** | **Enrolment^*^** | **CSF Aβ +ve ^^^** | **Enrolment^*^** | **CSF Aβ +ve ^^^** | **Enrolment^*^** | **CSF Aβ +ve ^^^** | **Enrolment^*^** | **CSF Aβ +ve ^^^** |
| **Age** | 0.54  (0.45-0.64) | 0.69  (0.56-0.82) | 0.49  (0.42-0.56) | 0.52  (0.40-0.64) | 0.55  (0.49-0.61) | 0.74  (0.62-0.87) | 0.58  (0.50-0.65) | 0.57  (0.42-0.70) |
| **Male or Female** | 0.48  (0.41-0.56) | 0.62  (0.51-0.73) | 0.59  (0.53-0.64) | 0.59  (0.49-0.68) | 0.58  (0.51-0.63) | 0.54  (0.41-0.67) | 0.48  (0.42-0.55) | 0.60  (0.47-0.70) |
| **Education level** | 0.46  (0.37-0.54) | 0.54  (0.41-0.66) | 0.46  (0.40-0.51) | 0.51  (0.41-0.61) | 0.41  (0.35-0.47) | 0.56  (0.42-0.68) | 0.48  (0.43-0.53) | 0.53  (0.42-0.62) |
| ***APOE* ɛ4 Genotype** | NA | 0.68  (0.56-0.79) | 0.55  (0.49-0.61) | 0.57  (0.47-0.66) | 0.54  (0.48-0.60) | 0.72  (0.60-0.83) | 0.49  (0.42-0.56) | 0.64  (0.52-0.75) |
| **Yes/No Family history for Dementia** | 0.50  (0.42-0.57) | 0.51  (0.39-0.63) | 0.50  (0.47-0.52) | 0.55  (0.52-0.58) | 0.40  (0.36-0.46) | 0.62  (0.49-0.75) | 0.56  (0.41-0.54) | 0.57  (0.46-0.68) |
| **Yes/No Subjective Cognitive Decline** | 0.42  (0.36-0.48) | 0.58  (0.48-0.67) | 0.49  (0.44-0.53) | 0.51  (0.44-0.58) | NA | NA | 0.49  (0.43-0.54) | 0.57  (0.45-0.67) |
| **Yes/No Low Memory Score** | 0.54  (0.39-0.55) | 0.76  (0.56-0.81) | 0.50  (0.44-0.55) | 0.53  (0.44-0.62) | 0.47  (0.43-0.53) | 0.50  (0.39-0.62) | 0.50  (0.44-0.55) | 0.52  (0.44-0.60) |

Legend: CSF = cerebrospinal fluid. Univariate analysis AUCs calculated with pROC-package in R of glm models (family=binominal, with DeLong confidence intervals). EPAD-LCS = EPAD longitudinal cohort study (trial-ready cohort). APOE = Apolipoprotein E gene. Shown effect sizes are: Age per 5 years older at baseline, APOE ɛ4 in contrast to no APOE ɛ4 genotype, male in contrast to female, highly educated in contrast to low or normal level educated, family history for dementia positive in contrast to family history for dementia reported.

**Table S3. AUC on multivariate model figure 2**

|  | **Multivariate model figure 2.** |  |
| --- | --- | --- |
| **Cohort** | **Enrolment (AUC)** | **Decreased CSF Aβ +ve^ (AUC)** |
| **Toulouse Registry*** | 0.57 (0.47-0.67) | 0.77 (0.65-0.89) |
| **ALFA Study** | 0.62 (0.55-0.68) | 0.66 (0.55-0.77) |
| **Generation Scotland** | 0.70 (0.64-0.76) | 0.88 (0.79-0.96) |
| **pilotHO.nl** | 0.63 (0.54-0.71) | 0.71 (0.58-0.84) |

Models included: Multivariate AUCs calculated with pROC package in R of glm models (family=binominal, with DeLong confidence intervals). CSF = cerebrospinal fluid. Age at baseline, APOE ɛ4 status, gender, highly educated in contrast to low or normal level educated, status on family history for dementia. * No APOE genotype included in enrolment analysis.
